# A Comparison between Endostatin and Conventional Biomarkers on 30-Day Mortality and Renal Replacement Therapy in Unselected Intensive Care Patients

**DOI:** 10.3390/biomedicines9111603

**Published:** 2021-11-03

**Authors:** Toralph Ruge, Anders Larsson, Miklós Lipcsey, Jonas Tydén, Joakim Johansson, Mats Eriksson

**Affiliations:** 1 Department of Emergency and Internal Medicine, Skåne University Hospital, 214 28 Malmö, Sweden; toralph.ruge@med.lu.se; 2 Department of Clinical Sciences Malmö, Lund University, 214 28 Malmö, Sweden; 3 Department of Medical Sciences, Clinical Chemistry, Uppsala University, 751 85 Uppsala, Sweden; Anders.larsson@akademiska.se; 4 Department of Surgical Sciences, Anaesthesiology and Intensive Care Medicine, Uppsala University, 751 85 Uppsala, Sweden; Miklos.Lipcsey@surgsci.uu.se; 5 Department of Surgical and Perioperative Sciences, Anaesthesiology and Critical Care Medicine (Östersund), Umeå University, 901 87 Umeå, Sweden; jonas.tyden@regionjh.se (J.T.); joakim.johansson@regionjh.se (J.J.)

**Keywords:** acute kidney injury, critical illness, endostatin, epidemiology, intensive care unit, mortality, renal replacement therapy, SAPS3

## Abstract

Endostatin may predict mortality and kidney impairment in general populations as well as in critically ill patients. We decided to explore the possible role of endostatin as a predictor of 30-day mortality, acute kidney injury (AKI), and renal replacement therapy (RRT) in a cohort of unselected intensive care unit (ICU) patients. Endostatin and creatinine in plasma were analyzed and SAPS3 was determined in 278 patients on ICU arrival at admission to a Swedish medium-sized hospital. SAPS3 had the highest predictive value, 0.85 (95% C.I.: 0.8–0.90), for 30-day mortality. Endostatin, in combination with age, predicted 30-day mortality by 0.76 (95% C.I.: 0.70–0.82). Endostatin, together with age and creatinine, predicted AKI with 0.87 (95% C.I.: 0.83–0.91). Endostatin predicted AKI with [0.68 (0.62–0.74)]. Endostatin predicted RRT, either alone [0.82 (95% C.I.: 0.72–0.91)] or together with age [0.81 (95% C.I.: 0.71–0.91)]. The predicted risk for 30-day mortality, AKI, or RRT during the ICU stay, predicted by plasma endostatin, was not influenced by age. Compared to the complex severity score SAPS3, circulating endostatin, combined with age, offers an easily managed option to predict 30-day mortality. Additionally, circulating endostatin combined with creatinine was closely associated with AKI development.

## 1. Introduction

Patients with multiple organ failure are often treated in intensive care. Although the support of reversibly failing organs is of crucial importance, knowledge on predictive variables facilitating goal-directed therapeutic interventions aiming to avoid irreversible organ damage is still limited. The mortality rate in the general intensive care population is high and any possibility to identify patients at risk of developing organ failure, either temporary or definite, might have the ability to counteract severe and prolonged morbidity as well as mortality. Furthermore, biomarkers could aid clinicians in such decisions, but no biomarker has excellent discrimination capability in intensive care patients.

Endostatin is an endogenous inhibitor of angiogenesis. The protein has a molecular weight of approximately 20 kDa and is derived from type XVIII collagen by proteolytic cleavage within its C-terminal end [1]. A study utilizing epitope-defined monoclonal antibodies has shown that glomeruli expressed collagen XVIII in their basement membranes [2]. Acute renal failure induced by ischaemia/reperfusion causes the expression of both endostatin mRNA and the protein itself [3]. Furthermore, local synthesis of endostatin in the ischemic kidney suggests its role in the basement membrane cell survival [4].

Apart from serving as a predictor, endostatin may also have therapeutic properties. In a septic mouse model, treatment with endostatin attenuated the inflammatory response and improved survival in a dose- and time-dependent way [5]. 

Against this background, the primary aim of this study was, therefore, to further elucidate the role of circulating endostatin in an unselected cohort of patients admitted to an ICU in a Swedish county hospital, which may differ from patients found in a tertiary care hospital. The main outcomes were short-term mortality (30-day mortality), AKI, and a need for renal replacement therapy. For comparison, we also analyzed SAPS3, a well-accepted but also complex severity score in ICU patients.

## 2. Materials and Methods

### 2.1. Study Population

All patients (mean age: 66 years; range: 23–96 years; 34% female (28% sepsis (SAPS3 score: 63 (41–105); 12% trauma (SAPS3 score: 47 (29–74); 30% internal medical (SAPS3 score: 52 (30–85); 30% miscellaneous (SAPS3 score: 60 (32–108))) admitted to the intensive care unit (ICU) at Östersund County Hospital, Sweden, between 1 February 2012 and 31 January 2013 were screened for inclusion in this study, which was approved (Dnr 2018/16–32) by the regional ethical review board at Linköping University. Since several of these, namely the severely ill patients, were unable to provide written consent, oral consent was considered sufficient. This was approved by the ethical review board and also adopted in cases where relatives gave their consent to study participation. The study, which was conducted in accordance with the 1964 Declaration of Helsinki and its subsequent amendments, was reported according to the STROBE guidelines.

We performed a post-hoc study on a prospective observational study. After obtaining consent, blood samples were collected and clinical data were registered for prospective analysis. Otherwise, no interventional procedures associated with this study were undertaken. A total of 278 patients were included. Demography of these patients has previously been published [6].

Inclusion criteria were:

- Admission to the ICU at Östersund hospital;
- Documented informed consent verbally approved by the patient or trustee;
- Need for arterial blood sampling and/or monitoring;
- Age of 18 years or above.

According to the ICU routines of Östersund County Hospital, Simplified Acute Physiology Score 3 (SAPS3) [7] and daily Sequential Organ Failure Assessment (SOFA) scores were recorded. Patients with acute kidney injury (AKI) were identified as previously described [8]. 

### 2.2. Blood Sampling and Analyses

Blood samples were drawn at ICU admission, collected in vacutainer tubes containing ethylenediaminetetraacetic acid (EDTA) and centrifuged, frozen, and stored at −80 °C until analyzed.

Plasma endostatin levels were determined in 2020 using a human Endostatin sandwich immunoassay (DY1098, R&D Systems, Minneapolis, MN, USA) according to the manufacturer’s instructions. The samples were analyzed as singletons and the standards in duplicates. The total coefficient of variation (CV) for the endostatin assay was approximately 5%. We used an initial sample dilution of 1:40 in 1% bovine serum albumin solution according to the recommendations of the manufacturer. Values above the standard curve were then rediluted and analyzed in a higher dilution. Within-run coefficients of variation for diluted samples analyzed in triplicates are typically below 5%. The lowest standard point for the endostatin assay was 62.5 pg × L^−1^. Endostatin results were not available during the time the patient was in the ICU. 

Plasma creatinine was analyzed on a Mindray BS380 (Mindray, Shenzhen, China) using IDMS-calibrated enzymatic creatinine reagents (8L24-31, Abbott Laboratories, Abbott Park, IL, USA). The endostatin and creatinine assays were performed blinded without knowledge of clinical outcomes.

### 2.3. Statistics

Data are presented as median (interquartile range; IQR). A Mann–Whitney U Test was used for intergroup differences. To describe the predictive value of plasma endostatin, plasma creatinine, SAPS3, SOFA and age were used; for AKI, RRT and death at 30 days and their corresponding 95% confidence intervals (95% C.I.) were used. The area under the curve was obtained from the receiver operating characteristic curve (AUC-ROC). Age was used as a surrogate for co-morbidities and an inherent risk of death. The best cut-off was defined at the maximal distance from the receiver operating characteristic curve and the diagonal. The prediction of the risk of death at 30 days, AKI, and RRT during the ICU stay with increasing endostatin levels, also with an adjustment for age, were calculated and presented as odds ratios. STATISTICA software, version 13.2 (StatSoft, Tulsa, OK, USA) was used for calculations. *p* < 0.05 was considered significant.

## 3. Results

### Study Cohort

As shown in Table 1, non-survivors were older, had an increased SAPS3 score, and had increased concentrations of creatinine and circulating endostatin compared to survivors.

Patients who developed AKI within seven days had increased levels of both endostatin and creatinine but were of a similar age and suffered a similar critical illness compared to other patients. 

**Figure 1:**
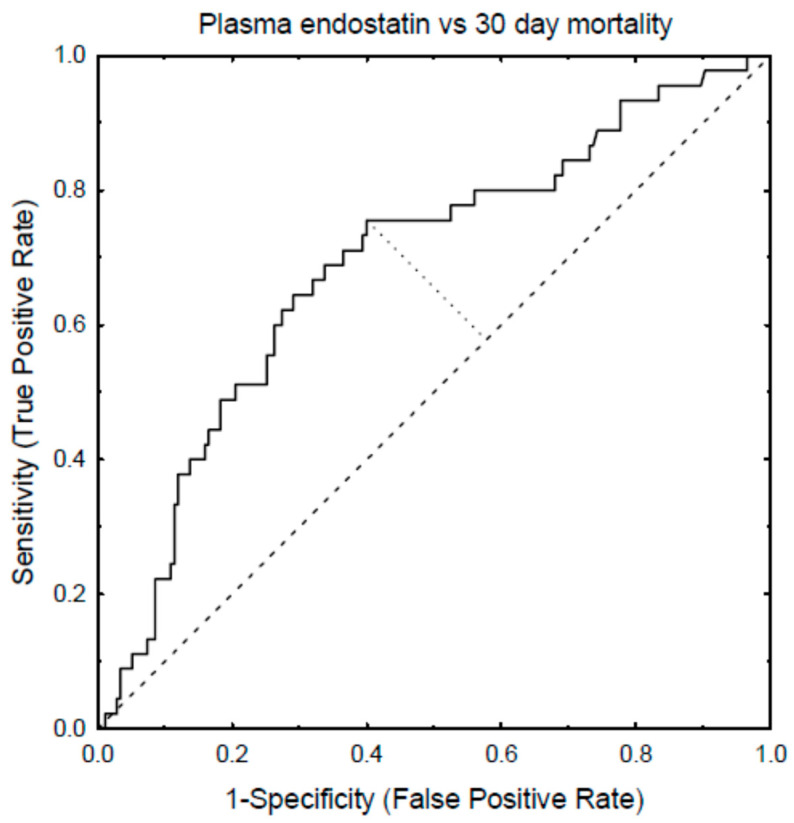
The receiver operating characteristic curve (AUC-ROC) depicting the plasma levels of endostatin, 30-day ICU mortality, and the determination of the cut-off value for endostatin on mortality.

AUC-ROC analyses showed that SAPS3 had the highest predictive value, 0.85 (95% C.I.: 0.8–0.90), for 30-day mortality (Table 2). Age in combination with endostatin, as well as age together with endostatin and creatinine, predicted 30-day mortality by 0.76 (95% C.I.: 0.70–0.82) (Table 3). Moreover, endostatin predicted AKI with 0.68 (0.62–0.74), and together with age and creatinine, with 0.87 (95% C.I.: 0.83–0.91). Endostatin predicted RRT, either alone (0.82 [95% C.I.: 0.72–0.91)), or together with age (0.81 (95% C.I.: 0.71–0.91)). Creatinine was, by itself, able to predict RRT (0.91 (95% C.I.: 0.85–0.97)). Endostatin was a predictor of mortality, AKI, and RRT in the univariate analysis (Table 4) and also with an adjustment for age (Figure 1). 

**Figure 2:**
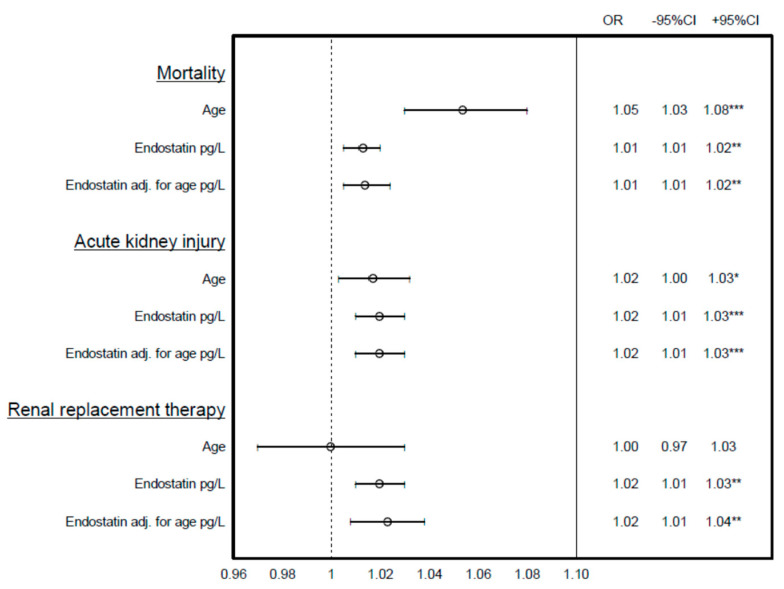
The risk of death at 30 days after ICU admission, acute kidney injury (AKI), and renal replacement therapy (RRT) during the ICU stay predicted by age, plasma endostatin, and plasma endostatin adjusted for age expressed as odd ratios (OR) with 95% confidence intervals (95%I). * *p* < 0.05, ** *p* < 0.01, *** *p* < 0.001.

In order to determine the endostatin levels that discriminate positive predicted outcomes from negative predicted outcomes, the cut-off levels were calculated and estimated to be as follows: the endostatin cut-off levels for AKI were 48 ng × L^−1^ (85 ng × L^−1^ for 90% specificity, and 26 ng × L^−1^ regarding 90% sensitivity), the cut-off level for endostatin to predict 30-day mortality was 47 ng × L^−1^, and the cut-off level for RRT was 62 ng × L^−1^ (Figure 2).

When endostatin was combined with other variables, such as the different age quartiles, an AKI of grade 0–3, and a SOFA score of 0–4, no striking differences in predictive values were noted (data not shown). In patients with sepsis, creatinine was a highly significant marker of AKI (*p* < 0.001), followed by endostatin (*p* = 0.001). There was no difference in SAPS3 or age in septic patients regarding AKI. There were no significant differences in age, SAPS3, creatinine or endostatin. Endostatin and age predicted an upcoming acute respiratory distress syndrome to the same degree (0.6 for both), and also the development of sepsis to the same extent (0.6 for both). When endostatin was combined with other variables, such as the different age quartiles, an AKI of grade 0–3, and a SOFA score of 0–4, no striking differences in predictive values were noted (data not shown).

## 4. Discussion

Plasma endostatin is a very promising marker for the prediction of AKI with a superior predictive value to, for instance, cystatin C and neutrophil gelatinase-associated lipocalin [9,10]. Endostatin, measured in 1112 patients in 17 ICUs in Finland, seemed to have limited value as a predictor of acute kidney injury (AKI), renal replacement therapy (RRT), and 90-day mortality [11]. However, in a large university hospital ICU setting, endostatin improved AKI prediction based on clinical risk factors [9]. The role of circulating endostatin is not clear. Circulating endostatin is an independent predictor of chronic kidney disease [12], as well as a predictor of long-term mortality in the general population [13]. Additionally, recently performed studies have shown a distinct association between high endostatin levels and indices of cardiovascular disease [14,15]. The role of circulating endostatin in critical ill patients has been sparsely studied. In patients admitted to the emergency department with acute dyspnea, endostatin was closely associated with 30-day mortality and was a better predictor of short-term mortality compared to the currently used medical triage tool. In the ICU setting, circulating endostatin has been shown to predict 90-day mortality, AKI, and renal replacement therapy (RRT) [9,11].

Collagen XVIII is widely present in the renal tubular epithelium, glomerular basal membrane, and Bowman’s capsule surrounding the glomerulus [16,17]. When the kidneys are injured, collagen XVIII is degraded, and endostatin is derived from the cleavage of collagen XVIII. Thus, the elevated plasma endostatin levels are derived from the kidney injury and the levels are associated with the degree of AKI [18].

Already mild AKI is associated with increased morbidity and mortality, and the risk increases with the degree of AKI. Patients with more severe AKI often require dialysis during their ICU time [19]. In most cases, there is an improvement of renal function after AKI, but many AKI patients will have persistent kidney dysfunction and may require dialysis for the rest of their lives, or kidney transplantations [20]. 

Connective tissues throughout the body contain collagens, which are widely expressed, being the most abundant protein providing structural support in the extracellular matrix (ECM). Collagens are not only a scaffold, which supports and protects the surrounding cells, but collagens are also deeply involved in physiological and pathological processes affecting organ function, e.g., intercellular communication [21]. The remodeling of an imbalanced ECM, which occurs during certain pathological conditions, leads to an expression of ECM fragments which have signaling properties, i.e., negative feedback loops on certain cells (e.g., endostatin having such effects on endothelial cells). When the inhibition of such feedback occurs, the net response may be an invasion of endothelial cells into the basement membrane, which may be favorable during the process of acute tissue injury repair, but in the long run may cause a dysregulated response characterized by a loss of original tissue function [22].

This investigation showed similar predictive values on mortality at 30 days after an admission to ICU, AKI within 7 days after ICU admission, and RRT by endostatin, age, and SAPS3. Increases in both endostatin, SAPS3, creatinine and age were found in patients that died within 30 days after ICU admission. Creatinine had a higher predictive value for AKI and RRT than endostatin and SAPS3. This is not unexpected, since the creatinine values were available during the patients’ care in the ICU and the definition of AKI is based on creatinine. Similarly, the decision to implement RRT is influenced by plasma creatinine levels. It should also be remembered that SAPS3 is partially based on the plasma creatinine level [1]. 

The median plasma level of endostatin in non-survivors was 67.6 nanog x L-1, suggesting that this value may be regarded as a signum malum, at which an offensive strategy against further deterioration should be implemented. Such strategies may include conventional ICU care, inotropy, optimizing ventilation, adequate anti-infectious treatment, and readiness for RRT. Intensified monitoring, e.g., lactate-guided therapy [23], which has been shown to reduce hospital mortality, may turn out to be of synergistic value when combined with further determinations of plasma endostatin. Thus, our findings may contribute to increased knowledge on the paradigm of antiangiogenesis on mortality as well as AKI, since higher postoperative levels of the antiangiogenic protein VEGFR1 in plasma were associated with a higher risk for AKI and mortality in patients undergoing cardiac surgery [24]. In contrast, serum endostatin 30 days after surgery was significantly reduced, compared to presurgical levels, in patients undergoing orthopedic and cardiopulmonary surgeries. Such a reduction would possibly favor angiogenesis and thereby wound healing [25]. This study is in agreement with previous ones [9,26], suggesting a role of endostatin as a predictor of death, especially when age and creatinine levels are taken into account, as well as AKI. The creatinine value is an important part of the decision regarding RRT. As the creatinine values, in contrast to endostatin, were available when deciding about RRT, this could have contributed to the high AUC for creatinine for RRT. 

A problem with endostatin, and many other ELISA methods, is the lack of international calibrators. Thus, each manufacturer has developed their own unique calibrator. Additionally, the immunogens, calibrators and controls in cytokine assays are usually recombinant. If these proteins are produced in bacteria, the post-translational modifications (e.g., glycosylations) will differ from the forms found in patient samples [27]. Depending on the antibody binding site of the specific antibodies used in an assay, this may cause additional intra-manufacturer assay variation. It is thus very complex to compare assay results from different assays. We have, therefore, used the same assay for more than ten years. We have also tried to standardize the dilutions. 

No lifestyle information from the patients were collected as it was considered not possible to collect reliable data on, e.g., smoking habits and physical activity as part of the ICU care. In several cases, relatives gave their consent to study participation. Thus, effects of lifestyle influences on endostatin levels and outcomes in this cohort is, unfortunately, lacking.

This study has some limitations. Conventionally, survival analysis is performed using Kaplan–Meier curves. However, we wanted to explore 30-day mortality and not the time from admission to death. Hypothetically, race or ethnicity could influence our results, since such variables are known to be confounding factors when endostatin is evaluated [28]. However, such a bias seems unlikely in our cohort, since this investigation was performed in a city in Northern Sweden, with a very homogenous population. Furthermore, the investigation was performed in a single center and therefore our results may not necessarily be possible to generally extrapolate. Another drawback is the fact that several years elapsed between sampling and analysis. Even though the samples were stored at −80 °C, a possible effect of ageing cannot be excluded. A limitation of the cut-off levels used in this study is the fact that the application of such a level segregates a test result from being either negative or positive, which may be mathematically, but not necessarily biologically, correct. The fact that both creatinine and SAPS3 results were available during the ICU period is likely to have improved their predictive value in relation to the other markers and hence underestimated the effect size of the predictive value of endostatin. This is a negative consequence of comparing blinded versus unblinded data, but from an ethical perspective it is the only option.

## 5. Conclusions

Endostatin seems to have some overall predictive value on the triad of 30-day mortality, the development of AKI, and the need for RRT. Creatinine, and thereby SAPS3, are also not precise in predicting outcomes. From this perspective, any tool that may help to steer therapy should be impartially evaluated. Future studies on endostatin may focus on the progress of this variable during stays in the ICU, and also on any interconnection with metabolic complications to severe disease. 

## Figures and Tables

**Table 1:** Levels of SAPS3, age (years), creatinine (micromol × L^−1^) and endostatin (nanog × L^−1^) on the day of admission in patients ceased and not ceased 30 days after admission.

	Survivors		Non-Survivors		*p*-Value
	Medican	IQR	Median	IQR	
SAPS3	53	45–63	72	66–79	*p* < 0.001
Age	64	49–79	72	68–79	*p* < 0.001
Creatinine	80	61–115	109	75–164	*p* < 0.001
Endostatin	39.8	27.8–63.2	67.6	47.4–94.4	*p* < 0.001

**Table 2:** Levels of SAPS3, age (years), creatinine (micromol × L^−1^) and endostatin (nanog × L^−1^) on the day of admission in relation to the development of acute kidney injury (AKI) seven days after ICU admission. n.s. denotes not significant.

	Non-AKI		AKI Day 7		*p*-Value
	Median	IQR	Median	IQR	
SAPS3	55	45–66	64	53–74	*p* < 0.001
Age	66	49–76	70	62–76	n.s.
Creatinine	81	60–92	132	107–186	*p* < 0.001
Endostatin	38.4	27.4–59.2	60.5	35.7–92.3	*p* < 0.001

**Table 3:** Predictive values of: death at 30 days after ICU admission; acute kidney injury (AKI); and renal replacement therapy (RRT) during the ICU stay. Values represent the area under the curve from the receiver operating characteristic curve (AUC-ROC (values within brackets are 95% confidence intervals)). N.A. denotes that calculation is not applicable due to few subjects.

Predictive Variable	30-Day Mortality	AKI	RRT
Endostatin	0.69 (0.62–0.77)	0.68 (0.62–0.74)	0.82 (0.72–0.91)
SAPS3	0.85 (0.80–0.90)	0.65 (0.58–0.72)	0.67 (0.52–0.82)
Age and Endostatin	0.76 (0.70–0.82)	0.70 (0.64–0.76)	0.81 (0.71–0.91)
Age, Endostatin and creatinine	0.76 (0.70–0.82)	0.87 (0.83–0.91)	N.A.
Age and creatinine	0.71 (0.64–0.78)	0.86 (0.82–0.90)	N.A.
Age	0.71 (0.64–0.77)	0.57 (0.50–0.64)	0.59 (0.42–0.76)
Creatinine	0.64 (0.57–0.71)	0.86 (0.82–0.90)	0.91 (0.85–0.97)

**Table 4:** SAPS3, age (years), creatinine (micromol × L^−1^) and endostatin (nanog × L^−1^) on the day of admission in relation to renal replacement therapy (RRT). n.s denotes not significant.

	Non-RRT		RRT		*p*-Value
	Median	IQR	Median	IQR	
SAPS3	58	48–69	71	56–75	n.s.
Age	68	54–76	66	53–72	n.s.
Creatinine	84	62–119	288	154–495	*p* < 0.001
Endostatin	45.4	28.6–67.4	87.3	68.3–144.2	0.002

## Data Availability

The datasets used and/or analyzed during the current study are available from the corresponding author on request. This will in most cases also require an ethical permit.
